# Infection control link nurses in acute care hospitals: a scoping review

**DOI:** 10.1186/s13756-019-0476-8

**Published:** 2019-01-28

**Authors:** Mireille Dekker, Irene P. Jongerden, Rosa van Mansfeld, Johannes C. F. Ket, Suzanne D. van der Werff, Christina M. J. E. Vandenbroucke-Grauls, Martine C. de Bruijne

**Affiliations:** 1 Amsterdam UMC, Vrije Universiteit Amsterdam, Department of Medical Microbiology and Infection Prevention, De Boelelaan 1118, room PK1X132, 1081 HV Amsterdam, The Netherlands; 2 Amsterdam UMC, Vrije Universiteit Amsterdam, Department of Public and Occupational Health, Amsterdam Public Health research institute, Amsterdam, The Netherlands; 3 Amsterdam UMC, Vrije Universiteit Amsterdam, Medical Library, Amsterdam, The Netherlands

**Keywords:** Liaison nurse, Nosocomial infections, Infection prevention and control, Infection control guidelines, Cross infection

## Abstract

**Background:**

Involving link nurses in infection prevention and control is a strategy to improve clinical practice that has been implemented in hospitals worldwide. However, little is known about the use, the range and benefits of this strategy. We aimed to identify key concepts of infection control link nurses (ICLN) and ICLN programs, to evaluate the effect of such programs, and to identify gaps in the evidence base.

**Methods:**

In a scoping review, we searched PubMed, CINAHL, Google and Google Scholar for manuscripts on ICLN in acute care hospitals. We included research- and opinion-based papers, abstracts, reports and guidelines.

**Results:**

We included 29 publications and identified three key concepts: the profile of ICLN, strategies to support ICLN, and the implementation of ICLN programs. The majority of included studies delineates the ICLN profile with accompanying roles, tasks and strategies to support ICLN, without a thorough evaluation of the implementation process or effects. Few studies report on the effect of ICLN programs in terms of patient outcomes or guideline adherence, with positive short term effects.

**Conclusion:**

This scoping review reveals a lack of robust evidence on the effectiveness of ICLN programs. Current best practice for an ICLN program includes a clear description of the ICLN profile, education on infection prevention topics as well as training in implementation skills, and support from the management at the ward and hospital level. Future research is needed to evaluate the effects of ICLN on clinical practice and to further develop ICLN programs for maximal impact.

**Electronic supplementary material:**

The online version of this article (10.1186/s13756-019-0476-8) contains supplementary material, which is available to authorized users.

## Background

Health care associated infections cause significant morbidity and mortality in patients and form a financial burden to health care systems [1], Appropriate application of universal precautions (e,g. hand hygiene) by health care workers has been proven effective in reducing transmission of microorganisms and subsequent acquisition of health care associated infections [2]. Still, in general, compliance with these simple infection control measures is low [3, 4].

A strategy to improve compliance is to involve dedicated nurses in infection prevention and control. Such dedicated nurses or infection control link nurses (ICLN) act as a link between their own clinical area and the infection control team and raise awareness of infection prevention and control. They are trained to educate colleagues and motivate staff to improve practice [5, 6]. Since their first introduction in the 1980’s, ICLN have been appointed in hospitals worldwide; they usually work within a hospital-based network [7–13]. The major investment in time and effort of the infection control team and link nurses that accompanies the implementation of an ICLN program is generally perceived as worthwhile [5, 14, 15].

An initial search for literature on ICLN and the interventions (e.g. programs) that are used to set up and maintain ICLN networks, however, revealed a lack of research on the effectiveness of ICLN in improving compliance with infection control guidelines or their impact on patient outcomes (e.g. health care associated infections) [16]. Before advocating ICLN programs, a better understanding of the use, range and benefits of these programs is needed.

A recent systematic review, focusing on facilitators and barriers of ICLN networks, included ten studies with a large variation in design and outcomes [17]. The authors searched only medical orientated databases, although the subject of study were nurses. Not searching nursing-orientated databases nor the grey literature in a relative unexplored field resulted in a small set of studies. To be able to assess all the available literature on link nurse programs in infection control in acute care hospitals we searched for studies published in different databases and in the grey literature. We looked at the key features of ICLN and ICLN programs, and aimed to evaluate the effects of such programs on awareness of infection prevention, guideline adherence and patient outcomes. Finally, we sought to identify gaps in the evidence base for ICLN networks, and opportunities for research.

## Methods

Scoping reviews are useful when available research is limited and heterogeneous in studies designs. They address broad questions and examine evidence regardless of study design [18–21]. The improved five-stage methodological framework of Arksey and O’Malley was used to structure this study [18, 20]. This entails an iterative technique of formulating and redefining the research question, identifying relevant studies, selecting studies, charting of the data, and collation, summarization and reporting of the results. As suggested by Daudt and Colquhoun, a quality assessment of the included studies was also performed [19, 21].

After the initial review of the literature the following research question was developed to guide the review: What is known about ICLN programs and their effectiveness in raising awareness of infection control or in the improvement of infection prevention practices, and do these programs reduce the risk of healthcare-associated infections?

Ebsco/Cumulative Index for Nursing and Allied Health Literature (CINAHL) and PubMed were explored on 18 July 2017 for index terms and text words with the initial search term “link nurs*”. Ebsco/CINAHL and PubMed were searched from inception up to 24 July 2017 (MD&JCFK). The following terms were used (including synonyms and closely related words) as index terms or free-text words: ‘link’ or ‘liaison’ or ‘intermediary’ and ‘nurses’ and ‘infection control’ or ‘handwashing’. Google and Google Scholar were searched for grey literature on 25 November 2017 and 8 February 2018. The search was updated on the 25^th^September 2018 (IJ&MD). The full search strategies for all resources can be found in the Additional file 1. Duplicate articles were excluded. The following criteria for inclusion were adopted: research- and opinion-based papers, abstracts, reports and guidelines, published between 1980 and 2018, specifically on infection control link nurses, and focused on acute care hospitals. Papers could be in the English, Dutch, German or French language. Studies investigating link nurses not specific to infection control or studies describing role models, e.g. ‘champions’, that led implementation of infection control guidelines were excluded from this review.

We retrieved full text articles that fulfilled the inclusion criteria outlined above. Two reviewers (SW&MD, IJ&MD) independently selected eligible papers and hand-searched reference lists for additional papers. Inter-rater reliability was tested after screening titles/abstracts (Kappa = 0.6). Results were compared, and disagreements resolved by consensus. When full texts were not available, corresponding authors were contacted. Each step of the study selection was discussed within the study team.

Two team members (SW&MD, IJ&MD) independently extracted and charted data on a predefined data charting form on country, study design, setting, key findings, and outcomes relevant to our research question.

Themes emerging from the data were analyzed and discussed within the research team. Descriptive numerical and thematic analyses are presented as narrative summaries given the heterogeneity of the literature. This process followed the Preferred Reporting Items for Systematic reviews and Meta-Analyses extension for Scoping Reviews (PRISMA-ScR) [22].

## Results

**Fig. 1:**
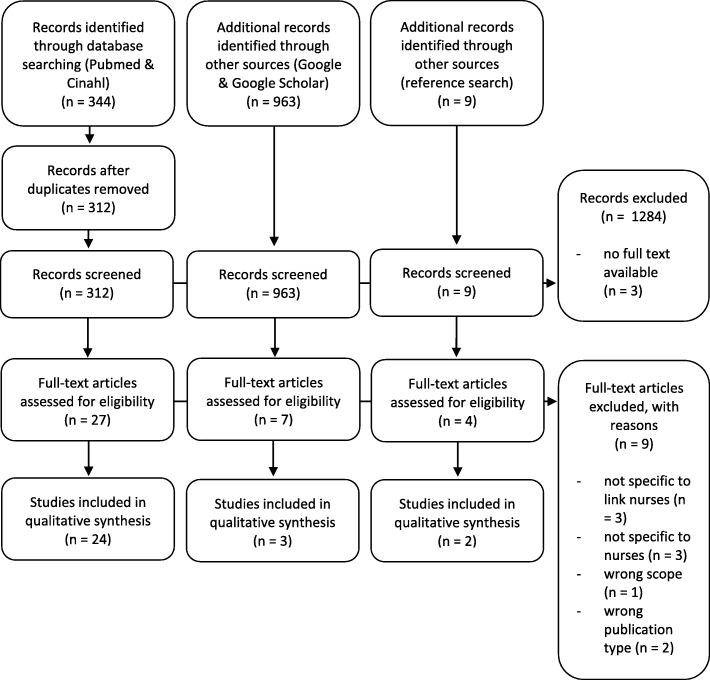
PRISMA flow diagram

Initially, we identified 312 articles in PubMed and CINAHL and additionally 963 papers in Google and Google Scholar. After screening for title and abstract, 36 articles were considered potentially relevant, of which 26 met our criteria. Hand searching reference lists identified 9 additional studies, of which 2 were included. One article was included after the last search update. In total 29 papers were included (Fig. 1).

The 29 included articles, 27 of which were peer reviewed papers, one guideline and one report represent literature from 5 continents. The majority of studies originated from the UK (*n* = 14). The other studies were conducted in the USA (*n* = 3), Australia (*n* = 2), China (n = 2), Japan (n = 2), Germany (n = 2), the Netherlands (*n* = 1), Egypt (n = 1), and Canada (n = 1). Belgian and UK researchers collaborated on one abstract. Most studies had a descriptive design (*n* = 12) or were before-after comparisons (*n* = 7). Other studies included qualitative studies (*n* = 4), cross sectional surveys (*n* = 2), studies using action research (n = 2), a mixed methods study (*n* = 1), and a randomized controlled trial (*n* = 1).

By charting the studies and summarizing the findings we identified that part of the studies focused on three major themes: the profile of ICLN, the implementation of ICLN programs, and strategies to support ICLN. The other part of the publications focused on outcomes of strategies that involve ICLN. Table 1 provides the details of studies including methodological comments and limitations of individual studies.

**Table 1:** Summary of included studies

Author details & Location	Study design	Setting	Key findings & Outcomes	Methodological comments & limitations
Braekeveld (2016) UK & Belgium	Abstract – interactive workshop and questionnaire on perception on the role of link nurses in infection prevention	450 voluntarily participants (link nurses, nurses, head nurses and infection control practitioners) in the UK and Belgium	A joint professional profile for infection control link nurses will follow	
Ching (1990) China	Cluster randomized controlled trial – introduction of a guideline for catheter care	1000 bed hospital in Hong Kong - Control group: three wards (surgical medical and gynecology) Test group: twenty-four wards	Three specific standards for urinary catheter care were significantly improved by link nurses educating their peers. Incorrect practices before intervention: - 63% intervention group - 68% control group (*p* = 0.4) Incorrect practices 5 weeks after intervention: - 36% intervention group - 48% control group (*p* < 0.05)	One hospital One baseline measurement No follow up Differing numbers in control and intervention wards (sampling bias)
Cooper (2001) UK	Descriptive paper- outline of the educational theory that underpinned infection control link nurses’ education	–	Education of ICLN should be based on educational theories.	
Cooper (2004) UK	Descriptive paper - prologue of action research study	A district general hospital	Methodological considerations and argumentation for action research.	
Cooper (2004) UK	Action research	A district general hospital - fourteen wards	Three out of four barriers for compliance with hand hygiene were significantly improved 3 months after intervention in 14 clinical areas	Small sample size No follow up
Cooper (2005) UK	Qualitative research - Focus group	Ten ICLN	ICLN reported increased feelings of empowerment, ownership and motivation during one focus group with 10 link nurses	No information on topic list, non- participants, number of data coders, data saturation, member check
Dawson (2003) UK	Narrative review - outline of the role of the ICLN	–	ICLN have a role in surveillance and education or peers. The role of the ICLN is still evolving. In 59% of National Health Services Trusts link nurses are active.	
Graaf de (2013) Netherlands	Descriptive paper – outline of the appointment of 8 link nurses to support the infection prevention and control team in a Dutch hospital	One hospital 8 link nurses	As a result of an outbreak 8 nurses were appointed ICLN They support the infection and prevention and control unit for 8 h a week and their departments are financially compensated	
Horton (1988) UK	Descriptive paper - outline of a pilot course	Sixteen ICLN in various services of a NHS trust	Monitoring performance of participants is crucial to the maintenance of high standards	
Jacobsen (1999) Australia	Descriptive paper –outline of an educational program / implementation strategy	560 bed adult teaching hospital - Operating Theatre	Isolation of the OT can make it more difficult for the ICN to encourage changes in infection control practice. ICLN can help to overcome this difficulty. Monitoring tools are necessary for long-term evaluation	–
Macduff (2009) UK	Full report - Evaluation of Cleanliness Champions Program using a mix of qualitative and quantitative methods	NHS health facilities in Scotland	Program has substantive positive influence on the prevention and control of health care associated infections in Scotland	No process or outcome measures (as guideline adherence or Healthcare Associated Infection rates stated) Perceived impact stated
Manley (2012) UK	NICE guideline - based on two workshops analyzed by an approach termed concept analysis	–	A national role profile and core competences to support link practitioners, their managers or organizations with a ICLN network	Consensus based guideline
Lene (2002) Australia	Descriptive paper – outline of structure and developments of a link program	A general acute care hospital	A program requires dedicated coordination, flexible and well planned education and effective support from management	
Lloyd-Smith (2014) Canada	Implementation of link nurse program, focus group & economic estimate evaluation	Three acute care hospitals - 16 clinical units 8 with link nurses 8 without link nurse	Seven link nurses produced an action plan. 10 focus groups with stakeholders led to 5 themes for a successful program Key factor is effective monitoring of effectiveness and sustainability The program was cost effective. (cost for link nurse program per bed ($490) vs cost for extra infection prevention practitioner per bed ($596))	Convenience sampling, no information on data saturation, no member check are risks for bias Important and relevant costs and consequences for each alternative were not identified
Millward (1993) UK	Cross-sectional - Audit tool & knowledge questionnaire	Three districts’ health authorities. One location with link nurse program.	Audits on eight infection control topics for 20 wards. Wards with infection control link nurses obtained higher scores on compliance with infection control standards (*p* = 0.0006). Link nurse showed higher scores on knowledge (69%) than non-link nurses (52%) (*p* = 0.008).	Sample sizes too small for analyses.
Miyachi (2007) Japan	Quasi experimental design	A 1133-bed University hospital	Significant decrease of monthly MRSA rates (from 6.3 to 5.0%) after implementation of link nurse system and during 2 year follow-up. Increase in monthly use of hand soap (17.3%).	As stated in article, risk of regression to the mean, maturation effects and confounding
Ross (1981) USA	Pre-post implementation study - establishing of ICLN on patient units	A 650-bed, university-affiliated general hospital	Implementation of ICLN and determination of health care associated infections rates in years one. Year two monitoring infection rates. Education met expectations of link nurses (96%). In 9 of 11 wards rates were reduced.	No baseline, no follow-up data.
Seto (2013) China	Before – after study & participatory action	A private 850-bed institution	Involving ICLN in brainstorm sessions, poster competition, identification of points of care and monitoring compliance improved hand hygiene practice significantly from 50 to 83%. Use of hand rub increased from 8.1 l/1000 patient days to 9.1 l/1000 patient days.	Single centered uncontrolled study, maturation effects
Shabam (2012) Egypt	Cross-sectional survey	Twenty hospitals, 205 head nurses who work as a ICLN in various departments (medical, surgical, neonatal, pediatric, obstetrics, gynecology, dialysis, outpatients’ clinics, emergency, burn and urology)	Survey results showed that ICLN have a role in education (25%), consultation (25%), administration (90%), research (21%) and supervision of safe practice (99%) The majority of head nurses participated in a training program related to infection prevention and control but not on their ICLN roles 48% of head nurses never performed ICLN roles. 54% had a low level of knowledge on infection prevention and control 79% had a high perception of infection prevention and control When head nurses’ knowledge and perception increased the performances on the 5 identified roles increased (*p* = 0.0001)	No description or definition of “perception as a link of infection control”
Sopirala (2014) USA	Quality improvement study (pre-post design)	A 1191-bed University Medical Center	After a 2 year baseline period link nurses were introduced during a year. In that year MRSA rates reduced (28%, p = < 0.01), MRSA bacteremia rates reduced (41%, *p* = 0.003), hand soap consumption increased(from 19 to 31 oz) as compliance with hand hygiene (from 30 to 93%).	No randomization, no follow-up
Sopirala (2018) USA	Before – after study evaluating a CAUTI prevention program with two different CAUTI definitions	A 699-bed tertiary care academic medical center	After a 21 month baseline period (data on urine cultures of 5 ICU units) link nurses were trained in CAUTI prevention, participated in training of colleagues and patients, and committed to ward based actions. CAUTI rates declined in with new definition (IRR 0.67, 95% CI [0.48–0.93]) CAUTI rates increased with old definition (IRR 1.12, 95% CI [0.88–1.43])	Single centered study, no follow-up
Teare (1996) UK	Interventions study - outlining how to design the ICLN network for the hospital	District general hospital	Implementation in 3 phases: set up, setting standards on wards, management ownership. Infection control practices were divided in 8 areas. ICLN (*n* = 51) had a role in education of peers and the audit of infection control practices. The link nurse system had a positive effect on clinical practices. Infection rates did not reduce. The infection control team was added to the trusts risk management group.	No baseline measurements, no follow-up. No exact numbers given.
Teare (1998) UK	Descriptive paper - reporting experiences and encountered benefits	Mid-Essex trust	Link nurses have a role in education and surveillance. ICLN system has raised awareness and increased the profile for infection control.	
Teare (2001) UK	Descriptive paper - outlining a study day for ICLN	Mid-Essex trust	Six interactive sessions on infection prevention knowledge and governance. A questionnaire quantified the self-assessed results of ICLN on their wards. This assessment of capabilities and limitations may be useful in the communication with ward management .	
Tebest (2017) Germany	Cross-sectional survey among ICLN (*n* = 64)	University hospital	Response rate 29% (*n* = 29). Intended services were rarely performed Barriers were the lack of release from other duties and the lack of acceptance of the role by physicians	One hospital Small sample
Tsuchida (2007) Japan	An intervention study with before and after comparison	560-bed acute hospital located in a major urban area in Japan	In year one risk factors for CLABSI in catheter care were identified with the help of 4 link nurses. In the following 2 years interventions were implemented. ICLN educated colleagues and observed catheter care. In those two years CLABSI rates declined from 4.0/1000 catheter days to 1.1/1000 catheter days (*p* < 0.005)	Single centered study, No randomization, no follow-up
Ward (2016) UK	Descriptive paper outlining the role of the link nurse	–	Currently there is limited evidence of the efficacy of ICLN in improving practice	
Wilbrandt (2001) Germany	prospective controlled study	Eight hospitals – four intervention and four controls	The concept of link nurses was introduced successfully. Improvements on the level of process quality (increase of contact moments between INLN and infection control staff) . No reduction of nosocomial infections.	No randomination Unclear duration of follow –up No definition for ‘success’ of the link nurses
Wright (2002) USA	Pre-post implementation observational study	A 87-bed neonatal intensive care unit at a Children’s hospital	Decrease of nosocomial infections The role of the ICLN is flexible and can be tailored to the specific needs	No N, percentage or 95%CI stated

### Key features

#### The profile of ICLN

Nine articles highlighted the ICLN profile with accompanying roles, tasks and competences [5, 6, 9, 13, 15, 23–26] using different terminology (e.g. roles vs tasks). ICLN were first described in 1981 as a liaison between the epidemiology department and clinical wards [9]. In the following years, the educational role was added [5, 14, 25]. The Royal College of Nursing published a national ICNL role profile for the UK in 2012. Four core themes were identified for the link nurse role: “act as a role model and visible advocate, enable individuals and teams to learn and develop infection prevention and control practice, act as a local communicator, and support in audit and surveillance” [12].

Tasks of the link nurse role that were considered viable included: perform surveillance of infections [9, 13, 15, 25, 26], monitor infection prevention and control practices [5, 9, 13], aid in the early detection of outbreaks of infection [5, 15, 26], improve clinical practice at ward level [5, 6, 13, 15, 23, 26], act as a role model [6, 23, 27], and assist in research [13, 26].The task of transferring information to peers and other healthcare staff is described in five articles [5, 13, 23, 25, 26]. One article states that the influence of ICLN might lay more in improving practice than in the dissemination of knowledge upon which these practices are based [5].

The core competences of ICNL for fulfilling these roles and tasks include: receptive for feedback, proactive, non-judgmental, approachable, resilient, authoritative, assertive and charismatic [5, 15, 24, 27]. Two out of five studies that describe the enrollment of ICLN stress the importance of voluntary registration. It is seen as an expression of motivation and enthusiasm for infection prevention and control, which are perceived as core competences for the uptake of the ICLN role [5, 23–25, 28]. Authority is perceived as essential for carrying out the role. Therefore clinically experienced nurses are preferred as ICLN [5, 24, 27]. The Royal College of Nursing summarized competences of ICLN as: “to be passionate about infection prevention and control, responsible for own actions, an active participant in the ICLN network, approachable, non-judgmental, inclusive, reflective, and respectful” [12].

#### Implementation of ICLN programs

Five papers describe operational barriers of implementing an ICLN program [5, 11, 16, 24, 29, 30]. Two papers report on ICLN programs that discontinued due to operational difficulties [5, 16]. ICLN struggle with low staffing and high workload leaving insufficient time for ICLN activities [5, 11, 24, 29, 30]. High staff turnover challenges hospitals to keep the number of trained ICLN up to standard [5, 24]. To overcome these operational barriers an ICLN program in a Dutch hospital was set up with only eight ICLN. These ICLN were exempted from duty eight hours a week in order to propagate infection control practices at the ward and hospital level [23].

The difficulties encountered by ICLN in their educational role are discussed in six studies [15, 24, 29–32]. Two studies noted that medical staff lacked acceptance of the role of the ICLN or the need for infection prevention and control practice [29, 30]. Jacobsen reports a lack of participation of medical staff in educational sessions by ICLN [32].

Three papers describe the presence of ICLN as a risk. Although visibility of ICLN in their role is perceived essential to trigger behavioral change, other health care workers may foster the idea that infection prevention and control is not their concern and rely on the ICLN for all infection prevention and control matters [15, 24, 31]. None of the studies provided clues or insights in what aspects of ICLN programs were most effective.

#### Strategies to support ICLN

Strategies to support ICLN were listed in 17 papers and include education, commitment and coordination by the infection prevention and control team, support from ward management, support from the senior hospital management, and support between ICLN themselves [5–11, 14, 23–25, 27–29, 31, 33, 34] Thirteen studies report on educational components of ICLN programs [5, 7–11, 14, 23, 24, 27, 28, 31, 34] The Scottish Government provides a national training to aid education [34]. Twelve studies report on a local educational program under the direction of the infection prevention and control team [5, 7–11, 14, 23, 24, 27, 28, 31]. It is advocated to underpin this program with theory on adult learning [31], engage in active learning forms [5], communicate on topics of interest prompted by ICLN themselves [7, 31] and to communicate on one topic per year to create focus [27]. There is a large variation in the content of these programs. The curricula include content related to knowledge of microbiology, modes of transmission, nosocomial infections, and infection prevention and control policies, the application of this knowledge in nursing practice, education in auditing and surveillance, and skills for the dissemination of this knowledge to peers [5, 10, 14, 23, 24, 31]. The latter is perceived as vital for ICLN to become effective role models [5, 14, 31]. In order to expand these skills experts (e.g. a psychologist) contributed to two programs to tutor on leadership and change-management skills [10, 24]. Four studies suggest an introduction course (range 1–10 days) [5, 7, 9, 10]. This introduction course could be given as e-learning, to permit ICLN to start their activities at any time at their own pace [5]. Four studies report on regular meetings with one to three months intervals [7, 10, 14, 27]. Education modes vary from interactive sessions [7, 14], lectures, tutorials [28] and visits to the Microbiology Laboratory [7], laundry services and sterile processing department [10],to self-learning packages [11] and sharing copies of relevant literature [29]. Lectures are repeated several times [7, 28] or held during (a provided) lunch to facilitate attendance [7, 15]. Support by the infection prevention and control team is described in five studies [6, 7, 10, 24, 25]. Supporting activities include providing ICLN promotional and educational materials [24], through newsletters, and by mentoring the ICLN through regular ward visits for the discussion of progress and current ward-based problems [7, 10]. Action research or brainstorm sessions are used to collaborate in research, for the development of an implementation program and for ward-based action plans or assignments [6–8, 10, 24].

Three studies describe the role of the ward management in the empowerment of ICLN in fulfilling their role [5, 9, 29]. This support can be promoted by referring other staff to ICLN, by scheduling infection prevention and control topics for discussion at ward meetings, and by allowing ICLN training time [5, 29]. Support of Senior ward management is described in three studies as enabling factor for the program as a whole [24, 25, 31]. Three studies describe networking between ICLN as a support mechanism. To create mutual communication, discussion and sharing of experiences with other ICLN is encouraged in regular meetings [24, 29, 33].

### The effect of ICLN programs

Five studies have evaluated the introduction of ICLN with respect to infection rates [7, 8, 26, 35, 36]. Two studies with a before-after design and one with a quasi-experimental design showed that the introduction of ICLN led to improved compliance with hand hygiene or increased hand soap / sanitizer consumption and a reduction of Methicillin-Resistant *Staphylococcus aureus* (MRSA) rates [7, 8, 35]. In two other studies ICLN achieved a reduction of CLABSI [36, 37]. In the USA the reduction of nosocomial infections in a neonatal intensive care unit was linked to the introduction of an ICLN [26].

In three studies clinical practices improved with the help of ICLN [28, 32, 38]. In a Hong Kong hospital ICLN improved the care for urinary catheters in a cluster randomized controlled trial. The second study demonstrated higher compliance rates with infection prevention policies on wards with ICLN [38]. The third study described improved compliance with standard precautions in an operating theatre with an ICLN. The role of the ICLN was perceived pivotal. Compliance was not reported on [32]. One paper described a positive effect of “raising the profile for infection prevention and control” [15]. Another study reported a perceived improvement of infection prevention and control practice [27]. Furthermore one study reported “an improvement at the level of process quality” in a general sense after the implementation of ICLN [33].

## Discussion

This scoping review revealed a lack of research evidence on the effects of infection control link nurses on guideline adherence and patient outcomes. The majority of included papers delineate the ICLN profile with accompanying roles, tasks and strategies to support ICLN without an evaluation of the implementation process or effects in clinical practice. Only two of these articles included a brief evaluation of the impact of their ICLN program on healthcare-associated infections [9, 26]. Therefore the value and impact of ICLN programs is difficult to assess [5, 39]. Studies that report on the effect of ICLN programs in terms of patient outcomes or guideline adherence describe positive short term effects. Several ICLN programs appeared to have discontinued, none of these studies, however, mentioned that they did so because of negative or no results [5, 16].

Six of the studies that did report on the effect of ICLN programs had a single-center uncontrolled study design [7, 8, 26, 35, 36, 38]. These studies hold a high risk of selection bias [40]. Prevention of healthcare-associated infections may be influenced by many other factors than the ICLN program itself, and controlled studies may not find significant effects due to low statistical power (type II error) [41]. The combination of study design and limited research output holds a risk for selective reporting of positive findings and publication bias. This might have influenced our findings.

The narrative synthesis is based on studies that vary in quality, design and outcome. We assessed study outcomes as having equal weight. Although standardized data extraction and an iterative team approach strengthened reliability, this may have led to bias in the categorization of our findings. Possibly, we missed relevant papers, since we chose to exclude studies on the role of champions and opinion leaders.

Although the quantity and quality of research on ICLN is limited, a common theme that emerges is that a number of factors are considered vital for the support of ICLN in the completion of their tasks. First of all educational programs are important. This is in line with previous findings that show that, to improve infection prevention practices education of health care workers is vital [42]. The content and delivery of education in ICLN programs is not standardized, but in general, education of ICLN by the infection prevention and control team to educate on infection prevention topics in regular meetings is considered best practice. This education can be extended by training in implementation skills by experts. With respect to how to set up educational meetings, focusing on one topic at each meeting is seen as important [27].

The ICLN profile is flexible and must be tailored to the local needs [5, 6, 39]. This is essential to facilitate nurses in the ownership of the ICLN role. A role profile clarifies expectations of ICLN for all stakeholders. It facilitates communication on the ICLN role and tasks within the organization [43].

Support by the management at ward level can empower ICLN to act as a role model and to disseminate knowledge to their peers. The adherence to guidelines will improve when management supports infection prevention and control measures [44] since this improves their leadership. De Bono et al. found an association between effective leadership and better adherence to infection prevention and control policies (e.g. hand hygiene and personal protective equipment) [45].

In the UK a generic role profile for ICLN is established by the Royal College of Nursing [12], but it is not clear in how many hospitals ICLN actually are appointed. ILCN are present in several hospitals throughout the Netherlands, but not everywhere [46]. In German acute care hospitals ILCN are mandatory [17]. Furthermore, link nurses have shown potential in other settings [47–51]. It is therefore justified to invest in further research.

There is a lack of studies that evaluate the process of implementation of ICLN and the outcomes of ICLN programs. Evaluation should consider how to tailor and deliver an ICLN program to maximize impact of link nurses on guideline adherence and patient outcomes. By assessing in which context which program has impact, research findings can help to tailor ICLN programs to the local situation [52]. An in-depth description on how ward management, the infection prevention and control team and the ICLN interrelate can help understand how to support ICLN in fulfilling their tasks [53]. Damschroder et al. confirms the importance of cooperation between professionals from different disciplines to realize behavioral change [54].Information on the perception of link nurses and their peers on the role and the perceived effectiveness of their effort can contribute to this in depth description.

Interdisciplinary collaboration in infection control networks may help overcome resistance of other health care workers [11, 54]. In this respect, studies focusing on how to involve other health care workers in general, and physicians in particular are needed .

Finally, there is a research gap in how to sustain ICLN programs, and on their economic value. For further research, we advocate the use of mixed method designs, since the implementation of an ICLN network can be considered a complex intervention. By measuring structure and process outcomes, the implementation of the intervention can be monitored and evaluated. Qualitative designs can help to understand and explain these findings and link them to the context in which the implementation took place [55],

## Conclusion

There is a lack of robust evidence on the effectiveness of ICLN programs. Available studies have methodological issues, small sample size or lack the consideration of the implementation process or patient outcomes. This affects the transferability and generalizability of research findings. The impact of ICLN programs on patient outcomes is difficult to assess because these are influenced by many other factors. Therefore it is justified that future studies should focus on the effects of ICLN on surrogate end points such as awareness of healthcare-associated infections, knowledge of infection control, and guideline adherence. There is also a lack in the understanding of how ICLN can best be supported to disseminate knowledge and to create change sustainably. Future research on these support mechanisms and their contextual factors is needed to further develop ICLN programs for maximal impact.

## Additional file

Additional file 1: Full search strategies for all resources (DOCX 16 kb)

## Abbreviations

ICLN: Infection control link nurses
PRISMA-ScR: Preferred Reporting Items for Systematic reviews and Meta-Analyses extension for Scoping Reviews
