# Supplementary material for: Infection control link nurses in acute care hospitals: a scoping review
Source: Antimicrob Resist Infect Control. 2019 Jan 28;8:20. doi: 10.1186/s13756-019-0476-8 (PMC6348687; doi:10.1186/s13756-019-0476-8)
Supplement: Supplementary file 1 — Full search strategies for all resources (DOCX 16 kb) [file 13756_2019_476_MOESM1_ESM.docx]

**Additional information** Full search strategies for all resources **Scoping search

Search strategy for Ebsco/CINAHL (18 July 2017)**

| **Search** | **Query** | **Items found** |
| --- | --- | --- |
| **S1** | TI "link nurs*" OR AB "link nurs*" | **133** |

**Systematic search

Search strategy for Ebsco/CINAHL (24 July 2017)**MH = keywords
+ = keyword with explosion
TI = words in title
AB = words in abstract

| **Search** | **Query** | **Items found** |
| --- | --- | --- |
| **S9** | S4 OR S6 OR S8 | **102** |
| **S8** | S1 AND S2 AND S7 | **46** |
| **S7** | MH "Infection Preventionists" OR TI((infection* OR cleanliness) N3 champion*) OR AB((infection* OR cleanliness) N3 (champion* OR preventionist*) | **1,736** |
| **S6** | S3 AND S5 | **19** |
| **S5** | MH "Nurse Liaison" | **709** |
| **S4** | S1 AND S2 AND S3 | **91** |
| **S3** | MH "Infection Control+" OR MH "Handwashing+" OR TI(“infection prevention*” OR “infection control*” OR “crossinfection prevention*” OR “crossinfection control*” OR icln OR ipc OR handwash* OR hand wash* OR “hand hygien*” OR handhygien* OR disinfecti* OR “co wash*”) OR AB(“infection prevention*” OR “infection control*” OR “crossinfection prevention*” OR “crossinfection control*” OR icln OR ipc OR OR handwash* OR hand wash* OR “hand hygien*” OR handhygien* OR disinfecti* OR “co wash*”) | **49,477** |
| **S2** | TI(intermediair* OR liaison* OR link OR links) OR AB(intermediair* OR liaison* OR link OR links) | **26,316** |
| **S1** | MH "Nurses+" OR MH "Nurses by Educational Level+" OR MH "Nurses by Role+" OR MH "Advanced Practice Nurses+" OR MH "Pediatric Nurse Practitioners+" OR MH "Nurse Practitioners+" OR MH "Nurses by Specialty+" OR MH "Nurses, Other+" OR MH "Nurse Administrators+" OR MH "Nurse Consultants+" OR TI(nurse*) OR AB(nurse*) | **332,963** |

**Search strategy for PubMed (24 July 2017)**
[Mesh] = Medical subject headings
[tiab] = words in title OR abstract

| **Search** | **Query** | **Items found** |
| --- | --- | --- |
| **#1** | (("Nurses"[Mesh] OR "Nurse's Role"[Mesh] OR nurse*) AND (liaison*[tiab] OR intermediair*[tiab] OR link[tiab] OR links[tiab]) AND ("Infection Control"[Mesh] OR "Cross Infection/prevention and control"[Mesh] OR "Infection/prevention and control"[Mesh] OR "Hand Hygiene"[Mesh] OR infection preventi*[tiab] OR infection control*[tiab] OR crossinfection preventi*[tiab] OR crossinfection control*[tiab] OR hand hygien*[tiab] OR handhygien*[tiab] OR hand wash*[tiab] OR handwash*[tiab] OR disinfecti*[tiab] OR co wash*[tiab] OR infection prevention control*[tiab] OR infection prevention and control*[tiab] OR icln[tiab] OR ipc[tiab])) OR ((infection*[tiab] OR cleanliness[tiab]) AND champion*[tiab]) | **232** |

**Search strategy for Google Scholar/Google 2 November 2017**

(“link nurse”|”link nurses”) AND (~infection prevention|~infection control|~crossinfection prevention|~crossinfection control|icln|ipc|~handwashing|~hand washing|~hand hygiene|~handhygiene|~disinfection|~co washing)

Search strategy for Google Scholar 8 February 2018

*German*

(~liaison|~bindeglied) AND (~Krankenschwester|~pflegekräften|~hygienebeauftragten|~pfleger|~pflegerin) AND (~”infektion kontrolle”|~Kreuzinfektion|~Händewaschen|~Handhygiene|~desinfektion|~Infektionsverhütung|~Krankenhausinfektionen|~Desinfizieren)

*French*

(~liaison|~link) AND (~infirmiere|~championne) AND (~infection|~”laver les mains” |~hygieniste|~desinfection)

*Dutch*

(~aandachtsvelder|~contactpersonen|~kwaliteitsmedewerker) AND (~infectie|~infectiepreventie|~kruisinfecties|~”handen wassen”|~desinfectie) AND ~verpleegkundige AND ~ziekenhuis
